# Invisible and undervalued: A qualitative study of laboratory workers’ experiences and perceptions of laboratory strengthening in Sierra Leone

**DOI:** 10.4102/ajlm.v13i1.2292

**Published:** 2024-05-31

**Authors:** Mohamed B. Jalloh, Eva Vernooij, Alice Street

**Affiliations:** 1 Research Unit, Joint Medical Unit, Republic of Sierra Leone Armed Forces, Freetown, Sierra Leone; 2 Department of Community Health, Faculty of Clinical Sciences, College of Medicine and Allied Health Sciences, University of Sierra Leone, Freetown, Sierra Leone; 3 School of Social and Political Sciences, University of Edinburgh, Edinburgh, United Kingdom; 4 Department of Interdisciplinary Social Sciences, Faculty of Social and Behavioural Sciences, Utrecht University, Utrecht, the Netherlands

**Keywords:** Ebola outbreak, neglect, perceptions, investment, laboratory technicians, laboratory capacity, infrastructure

## Abstract

**Background:**

The 2013–2016 West Africa Ebola outbreak highlighted the importance of laboratory capacity to outbreak response while also revealing its long-standing neglect. The outbreak prompted massive international investment into strengthening laboratory services across multiple healthcare settings.

**Objective:**

In this article, we explore hospital-based clinical laboratory workers’ experiences and perceptions of their everyday working environment in Sierra Leone, and how recent investments in laboratory strengthening have shaped these.

**Methods:**

This qualitative study draws on in-depth interviews with eight laboratory workers and participant observation of laboratory practices at a tertiary referral hospital in Freetown between April 2019 and December 2019. Interview and observational data were coded and analysed using a reflexive thematic approach.

**Results:**

The Ebola outbreak prompted international investments in automated devices, biosafety training, and a new dedicated infectious diseases laboratory. However, little investment was made in the infrastructure and supply systems needed to sustain routine laboratory work or keep machines functioning. Laboratory workers perceived their work to be under-recognised and undervalued by the government, hospital managers and clinical staff, a perception compounded by under-use of the hospital’s laboratory services by clinicians.

**Conclusion:**

Understanding laboratory technicians’ views, experiences, and priorities is essential to any sustainable laboratory-strengthening effort. Investments in personnel should match investments in technologies and infrastructure for outbreak response.

**What this study adds:**

This study contributes to an understanding of how clinical laboratory personnel in Sierra Leone view and experience their work, and introduces the concept of social invisibility to explain these experiences.

## Introduction

The Ebola outbreak exposed significant gaps in Sierra Leone’s laboratory system and prompted a surge in international assistance for laboratory strengthening and emergency preparedness.^1,2,3^ During and after the outbreak, the public laboratory was a key target for interventions aimed at improving laboratory services, including training of personnel, provision of equipment and infrastructural improvements.^4,5,6^ Several publications have explored the experiences of international laboratory teams concerning their diagnostic achievements and laboratory support during and after the outbreak.^7,8,9,10,11,12,13^ Other publications have focused on the experiences of frontline health workers, including nurses and doctors.^14,15,16^ However, far less attention has been given to the West African laboratory workers’ experiences during Ebola and in its aftermath, or their priorities for improving routine and emergency clinical laboratory services.

Laboratory workers can provide unique insight into the changes to clinical laboratory work that have occurred since the Ebola outbreak, especially the impact of international assistance on everyday laboratory work in the country. The few studies published in this area point to the need for more in-depth qualitative research to understand the sociocultural and institutional contexts within which laboratory work becomes valued or devalued and in which careers are developed.^17,18,19^

This article aims to understand the experiences of laboratory workers in a government-funded military hospital in Freetown, Sierra Leone, their reflections on the Ebola outbreak, and their views on how management, government, and international partners can best support and improve their work. It contributes to a growing literature around laboratory strengthening in African countries,^1,2,19^ by providing on-the-ground perspectives from a country that has been the locus of intensive laboratory-strengthening interventions in a post-epidemic context. We argue that such efforts must consider the experiences and priorities of laboratory workers on the ground, since this group can best advise on the everyday realities of laboratory work and which interventions are most likely to improve morale, workflows, and capacity. We aim to generate new insights into everyday laboratory work that will be valuable to a growing range of laboratory-strengthening efforts in Africa, including those spearheaded by pan-African organisations such as the African Society for Laboratory Medicine and the Africa Centres for Disease Control and Prevention.

## Methods

### Ethical considerations

Ethical approval for the study was granted by the Sierra Leone Ethics and Scientific Review Committee (approval date 10 September 2018) and the University of Edinburgh Research Ethics and Integrity Committee. We also obtained approval from the 34 Military Hospital (MH 34) (Reference JMU/1000/1) where the data on which this article is based were collected. Formal written consent was obtained from all interview participants. To maintain patient confidentiality in the study, all identifying information was anonymized and replaced with unique codes and access to the data was restricted to authorized personnel only.

### Setting

This study forms one component of a larger research project on post-Ebola laboratory strengthening in Sierra Leone, funded by the European Research Council. The total research project included ethnographic research on laboratory work and clinical diagnostic pathways at a public referral hospital in Sierra Leone,^19,20^ a historical review of policy and planning relating to laboratory strengthening in Sierra Leone,^3,20^ ethnographic research on diagnostic processes and workflows at Community Health Centres,^20^ survey-based research on community-based fever management, and a district level survey of diagnostic availability and useability.^20,21^

This sub-study involved qualitative research at the 34 Military Hospital (MH 34), Freetown, Sierra Leone, which played a central role in the medical response to the Ebola outbreak. It was the first hospital to manage Ebola cases in Freetown and sent its health workers, including the first author of this article, to support several Ebola Treatment Centres and Community Care Centres across Sierra Leone. Its laboratory technicians were crucial in collecting and transporting samples from patients to designated laboratories to detect the Ebola virus. The MH 34 was also the site of intensive international laboratory-strengthening efforts during and after the epidemic, including the establishment of a Chinese government-funded infectious diseases laboratory.^9,22^

The MH 34 in Freetown, Sierra Leone, was established during British colonial rule to provide medical services to serving military personnel and their dependents. Currently, the hospital offers free care to military personnel and their dependents, while charging the public for services. The hospital’s core funding comes from the Sierra Leone government’s allocation to the Ministry of Defence. It, therefore, treats patients from across the spectrum of ethnic, religious, and economic groups in Sierra Leone. The MH 34 is the leading infectious disease facility in Sierra Leone for treating patients infected with high-consequence pathogens.^23^ It also hosts the country’s Rapid Deployable Isolation and Treatment Facility that responds to outbreaks in any part of Sierra Leone within 72 h to 96 h.

The MH 34 has two clinical laboratories: the main and infectious diseases laboratories. The main laboratory is as old as the hospital and includes phlebotomy, HIV/AIDS, serology, haematology, biochemistry, blood banking, and microbiology sections. It has 20 publicly employed personnel, and the government of Sierra Leone funds its equipment and consumables, with help from non-governmental organisations and international donors. Study participants were drawn from formally employed and volunteer laboratory technicians. Volunteers receive transport refund, are recruited by the hospital, and do not receive any salary. Paid staff, however, receive salaries and other benefits of employment and are only recruited by the Human Resources Management Officer of the government of Sierra Leone.

The infectious diseases laboratory is part of the Infectious Disease department built in 2018, with funding from the Government of the People’s Republic of China. The infectious disease laboratory provides advanced services in haematology, microbiology, and serology for patients admitted to the adjacent 30-bed infectious disease ward, performing routine clinical testing without a surveillance mandate. Staff members work in both the infectious disease laboratory and the main laboratory, and are rotated between the two, with three Sierra Leonean staff working in the infectious disease laboratory at any time. The infectious disease laboratory also has two permanently posted Chinese staff. Its equipment and consumables are funded and supplied by the Chinese military. In addition to these two clinical laboratories, a Biosafety Level 2 research laboratory, also built by the Chinese government in 2018, supports the prompt diagnosis of emerging high-consequence pathogens in Sierra Leone and conducts studies on infectious disease burden in the country.^23^

### Sample

Purposive sampling was used to ensure that Sierra Leonean laboratory workers with a wide range of demographic characteristics and employment experiences were included in the study. For selection, we categorised laboratory workers based on which clinical laboratory they worked in, which laboratory section they worked in, whether they were employed as payroll staff or as volunteers, and whether they worked on the laboratory bench or as a technical lead. We purposefully selected participants within each of these employment groups to ensure overall representation from a wide range of demographic groups, including gender, ethnicity, religion, and age. Sample size was determined by a combination of inclusion of participants from each of these groups and qualitative data saturation, defined as the point at which no new themes were emerging from the data.

### Data collection

The lead author, M.B.J., who is employed at the 34 Military Hospital as a physician, conducted data collection for this study between April 2019 and December 2019. His status as a hospital employee provided valuable insight into the institution’s historical and social context, and was essential to the ability to translate research findings rapidly into impactful institutional changes. During data collection, measures were implemented to minimise both participant and researcher bias. To minimise any potential for participant bias arising from M.B.J.’s employment in the hospital, participants were fully informed about the objectives of the study, namely to understand their experiences, efforts that would be made to anonymise their data, and limitations to these efforts given the small sample size, and their rights as research subjects. To mitigate researcher bias arising from M.B.J.’s prior knowledge of the institutions, co-authors were consulted regularly to obtain a perspective and ensure that M.B.J.’s biases and assumptions did not unduly influence the research process. Semi-structured interviews were conducted using a topic guide developed for the broader study and adapted for MH 34 by E.V., A.S., and M.B.J. The interviews focused on topics such as career history, experiences during the Ebola outbreak, daily workflows in the laboratory, changes in laboratory work over their careers, challenges faced in the laboratory, and priorities for improvement. The interviews took place in the interviewee’s workspace, ensuring privacy, and lasted an average of 40 min. Trained translators translated the transcripts from Krio to English. To verify the accuracy of the translation, M.B.J., who speaks Krio fluently, reviewed the English translations against the audio recordings for any unclear areas before approving the data for thematic analysis. All interviewees provided consent for audio recording. Ethnographic observation by M.B.J. was also conducted in the laboratory, by observing working practices, interactions, equipment usage, and the use of the workspace throughout the working day. These visits lasted between 2 h and 4 h, and occurred on different days and times to gather a comprehensive understanding of laboratory life. During these visits, M.B.J. also asked laboratory workers questions about their work, equipment, and infrastructure to uncover additional insights not covered in the formal interviews.

### Data analysis

A reflexive thematic analysis approach was employed to allow collaborative interpretation of the qualitative data and to identify patterns and themes across the data set.^24,25^ Due to the small sample size, this analysis was undertaken using Microsoft Word (Microsoft Corporation, Redmond, Washington, United States). The authors A.S. and M.B.J. each read all the interview transcripts and coded them. After that, a meeting was held with A.S., M.B.J. and E.V. to review the coding for each transcript, ensure consistency across the transcripts, check for researcher bias, and resolve any differences between the coders. Following a further review to check the coding schema’s consistency, coherence and logic, E.V., A.S., and M.B.J. collectively collated the codes into larger thematic groupings. The team then aligned individual codes, themes, and example quotations and cross-checked for overlaps between themes and the overall coherence of the thematic structure.

## Results

A total of eight laboratory staff participated in interviews for this study (Table 1). We interviewed six laboratory technicians who worked across all laboratory sub-departments. The two laboratory leads we interviewed had responsibility for managing the two clinical laboratories. Of the eight interview participants, only one was female, reflecting the broader demographics of laboratory staff, with 19 male and 2 female employees at the time of the study. All participants had experience working in an outbreak, and seven had direct experience working during the Ebola outbreak. All participants had diplomas in medical laboratory sciences at the Eastern Technical University of Sierra Leone.

**TABLE 1:** Characteristics of study participants at the 34 Military Hospital, Freetown, Sierra Leone, April 2019 to December 2019.

Participant characteristics	Frequency
Position	
Laboratory technician	6
Laboratory lead (manager)	2
Ethnicity	
Kissi	2
Mende	5
Mende-Madingo	1
Age (years)	
20–29	2
30–39	3
40–49	2
50–59	1
Religion	
Christianity	5
Islam	3
Gender	
Male	7
Female	1
Worked during the 2013–2016 West African Ebola outbreak	
Yes	7
No	1
Affiliation	
Soldier	5
Civilian	3
Employment status	
Employed	6
Volunteer	2
Working at	
Infectious disease laboratory	3†
Main laboratory	5‡

† , Participants 3, 6 and 8 worked at the infectious disease laboratory at the time of the study.

‡ , Participants 1, 2, 4, 5, and 7 worked at the main laboratory at the time of the study.

### Career experiences

Laboratory technicians were often encouraged by family members to pursue laboratory medicine:

‘My initial interest was in engineering science, but they [*mum and friends in healthcare*] unanimously encouraged me … to do laboratory medicine.’ (Participant 2, laboratory technician, 20 June 2019)

Three staff experienced financial difficulties after writing the national high school qualifying exams and felt pressured to study laboratory medicine as a route to economic security. Four opted to become laboratory technicians after volunteering or being employed as nurses or military officers for several years. At the time of data collection, one of the staff had volunteered for seven years and had not yet progressed to formal employment. Towards the end of the civil war, several programmes were introduced into the military to convert the fighters into professionals as a part of efforts supported by the British government, and some participants reported receiving laboratory training through these schemes.

For the few who had initially trained as nurses, their primary motivation to become laboratory technicians was that they would be able to support medical care for patients more effectively through laboratory work than through nursing:

‘[*S*]o I decided to enter the lab because … I saw that the laboratory is the basis of medicine because it really tells you what a person’s disease is, and when you know about a person’s disease, that’s when you’ll be able to fight it; if you don’t know what that person’s disease is and you give medication, that medicine will endanger that person’s health.’ (Participant 4, laboratory technician, 23 June 2019)

All but one of the technicians had experience working during the 2013–2016 West African Ebola outbreak, and, in many cases, this was described as a significant career moment that influenced later decisions and perceptions about laboratory work. Several technicians described their willingness to work during Ebola in terms of a sense of public duty. Many also described this as a period of self-sacrifice, which often involved significant personal costs regarding separation from relatives or the risks involved. Health workers known to have contact with Ebola patients and/or samples described being stigmatised by colleagues and the public. Two study participants stopped working as laboratory technicians during the Ebola outbreak. The first changed his role to a quarantine officer, while the second stopped working altogether because his boss died of Ebola, and his family insisted that he could not continue to work. Some interviewees also described this as a period of intensive training, which provided opportunities for learning new skills:

‘I had issues with my home because of my Ebola work, but I was determined to serve the people.’ (Participant 8, laboratory technician, 14 September 2019)

‘I was attached to the [*Ebola*] treatment centre that was managed by MSF [*Médecins Sans Frontières*]. I attended training, for which they gave me a certificate before I started working. We were trained on Infection prevention and control and the use of personal protective equipment [*PPE*] when collecting blood samples.’ (Participant 5, laboratory technician, 09 September 2019)

Some were motivated to continue their service as laboratory technicians, having seen the central role that laboratory medicine played in detecting and responding to outbreaks:

‘If we have another outbreak, no one will be able to detect the causative organism unless the laboratory technician runs the sample and provides answers as to what the causative organism is.’ (Participant 5, laboratory technician, 09 September 2019).

### Impact of Ebola outbreak on laboratory work

Participants described the 2013–2016 West African Ebola outbreak as highlighting the central role that the laboratory plays in clinical care. Participants described increased investment in high-end laboratory equipment in the main lab, especially automated machines, during the outbreak as one outcome of this increased visibility:

‘Specifically, the GeneXpert was brought here due to the Ebola.’ (Participant 7, laboratory lead, 12 September 2019)

Staff also received much training on biosafety, which improved practices like hand washing, waste segregation and use of gloves during sample collection, transportation, and analysis:

‘Before Ebola, some of us were even handling samples with bare hands because we didn’t believe in difficult infectious disease, we just thought of diseases like syphilis, gonorrhoea because it’s curable we didn’t take care too much. Because when we contract those types of diseases within a few days after taking treatment, it’s treated. In fact, I think it was Ebola that made the majority of people become aware of even RVS [*retroviral status*], which is HIV and AIDS and Hepatitis because they’re all viral diseases.’ (Participant 4, laboratory technician, 23 June 2019)

As Ebola outbreak-related work increased, most other patients avoided coming to public hospitals, which led to a decline in the routine functions of the laboratory. Unlike other components of laboratory medicine, little investment went into improving the existing hospital laboratory space and infrastructure where laboratory services are delivered. Participants pointed out that the main hospital laboratory did not undergo any renovation or expansion during or after the Ebola outbreak. A notable difference was the dedicated infectious diseases laboratory space built post-Ebola as part of a 30-bed Chinese government-sponsored Infectious Disease department. Participants described the infectious disease laboratory as having a ‘modern’ space, new equipment, a reliable supply of reagents and other utilities and a more comprehensive range of tests, and notably, a separate repair and maintenance system, financed and operated by the Chinese government, which was unavailable to the main laboratory, leading to regular machine breakdowns in the main laboratory, despite the investments in more high-end automated machines during Ebola:

‘At the other laboratory department, they don’t do biochemistry tests because of the machine [*breakdown*]. But here at the Infectious Disease department, we conduct all necessary tests precisely for patients.’ (Participant 6, laboratory technician, 11 September 2019)

### Training

Training was a recurrent theme in the interviews, including discussing positive training experiences and the need or desire for training in particular areas. Many participants positively described the additional training they had received during the Ebola outbreak. Despite this, they highlighted the need for additional and routine training in biosafety and equipment maintenance areas. Some hoped for additional professional training abroad, citing that overseas training is often more structured and that staff sent on such training would be more focused than when training is delivered locally:

‘We actually are expecting both international and national training. Specifically, we are out of someone who is trained in biomedical engineering. It’s really important, really important.’ (Participant 7, laboratory lead, 12 September 2019)

‘They just trained us to do the basic maintenance that is clean[*ing*] … we are not trained on that [*thorough check of equipment*]. So, we wait these people who we call from Guinea. They too call for American Embassy before ever they can even come and see about these machines.’ (Participant 1, laboratory technician, 07 June 2019)

Another participant suggested providing equipment and training to laboratory staff for fire prevention or management:

‘Well, to improve also the safety, safety talking about the security risk, the case of fire outbreak. Fire extinguishers, people are trained about fire safety. … Well, I say this because we do have experience with electrical problems.’ (Participant 7, laboratory lead, 12 September 2019)

### Laboratory infrastructure

The laboratory service at the time of the study was characterised by infrastructural challenges, such as erratic electricity supply, the absence of stabilisers, hot storage spaces for reagents, and a lack of maintenance for workspaces, leading to a short lifespan for laboratory equipment and supplies:

‘Even in our store here, you will realise upon entering that it is hot for you; what do you think its effect would be for supplies in cartons?’ (Participant 2, laboratory technician, 20 June 2019)

Failure to expand the laboratory infrastructure or renovate the existing spaces for over a decade despite the Ebola outbreak meant that staff, equipment, and the public were exposed to rain due to the leaking roof:

‘They only do the repainting and nothing more.’ (Participant 5, laboratory technician, 09 September 2019)

‘The building leaks, and in the rainy season, the machines get wet whenever it rains.’ (Participant 8, laboratory technician, 14 September 2019)

The absence of a biosafety cabinet and regular maintenance for laboratory equipment leaves laboratory staff vulnerable with a sense of neglect and marginalisation. At the time of the interview, there was no functioning biochemistry, haematology or hormone analyser in the main laboratory, and the staff interviewed pointed to infrastructural gaps as the primary cause for the breakdown of the analysers:

‘At the main laboratory, there are so many non-functional machines there. The building needs to be expanded.’ (Participant 8, laboratory technician, 14 September 2019)

‘Faulty equipment such as the haematology analyser or printer limit the quality of services that we provide … the building itself is not ideal; it is archaic now and too small for us.’ (Participant 2, laboratory technician, 20 June 2019)

Several automated machines in the laboratory were procured in the years following the Ebola outbreak, including a Sysmex haematology analyser, electrolyte analyser and electrophoresis machine. However, the Sysmex machine was used for less than a month before it broke down. The electrolyte analyser and electrophoresis machine were never fully installed. Altogether, three machines were not working at the time of the interview:

‘Yeah, we have, we have two, ok three, like even the Hb [*haemoglobin*] electrophoresis had the same problem. The haematology analyser has the same problem, and the Biochemistry analyser has the same problem. So, we have three machines that are down right now.’ (Participant 1, laboratory technician, 07 June 2019)

Staff contrasted the issues with physical infrastructure and maintenance of the main laboratory with the modern equipment and space at the infectious disease laboratory. Interviewees characterised the infectious disease laboratory in terms of regular maintenance routines and the exercise of quality controls that the laboratory staff were trained and supervised to perform by laboratory professionals deployed to Sierra Leone as part of the Chinese Military Medical Expert Group:

‘In the morning [*at the infectious disease laboratory*], I will have to clean the machines, cross-check the waste and distil [*sic*] water from the haematology machine. I will also check the printer to see if it’s functional. If there is a need to add or change the distilled water, I will do so afterwards. If there is a need to empty the waste, I have to do so to avoid the machine breakdown. I will also do the same in the Biochemistry department, checking the waste, the machine’s temperature, and the pins that help to operate the machine. I also need to check all the pins and needles of the Biochemistry machine to avoid breaking them. And quantity. If we run low on some reagents, I will mix more using the manuals provided.’ (Participant 6, laboratory technician, 11 September 2019)

### Supply chain

Equipment procurement was a sensitive topic, and some laboratory staff hesitated to discuss it. The interviewees reported little or no consultation on the quantity and type of equipment and reagents required by the lab. Some reported receiving reagents that did not match the equipment, leading to equipment breakdown:

‘We may receive a call, and at times our boss may not even be informed on the procurement or coming of the new machine and at times, they may even double a specific machine because they have failed to discuss the matter with us, and they know not exactly what the lab requires.’ (Participant 2, laboratory technician, 20 June 2019)

At the point of data collection, the laboratory had not received any reagents for several months. Stockouts of reagents were common at the main laboratory, leading to redundant equipment. The laboratory relies on only one vendor to supply reagents to the laboratory, and interviewees described this ‘monopoly’ as the cause of erratic supplies:

‘The government provides these supplies through a private contractor. The problem we have is that machines that are in frequent use receive little or no reagents, while we continue receiving reagents for machines that are faulty or dysfunctional. We, therefore, always encounter stockouts of reagents before another batch of supplies is received.’ (Participant 5, laboratory technician, 09 September 2019)

Some donor-supported tests, such as HIV tests, were also reported as unsustainable and afflicted by nationwide stockouts:

‘For instance, the cartridge for the GeneXpert machine ran out of stock for a long time and replenishing it may take up to 3 or 4 months. At times, there is stockout of such cartridges in the entire country.’ (Participant 2, laboratory technician, 20 June 2019)

The infectious diseases laboratory received equipment and reagents from the Chinese military and was reported as largely having a sustainable supply. Even so, interviewees reported that culture and sensitivity testing was not done at the infectious disease laboratory because the cassettes are expensive and unavailable in the local market.

### Workplace relationships

A major challenge highlighted by participants was staff shortages that resulted in a high daily workload and long turn-around times:

‘We actually don’t have enough staff, but those of us here, we are doing all we can to complete our task before the day ends. Most of the time, I don’t even return home. I will have to sleep in our department. So, at times, I will wake up at night and run some of the tests before daybreak.’ (Participant 6, laboratory technician, 11 September 2019)

The poor allocation of human and material resources to public clinical laboratories and the perceived lack of career opportunities in laboratory medicine is evidence to the staff that their work is undervalued by other clinical staff and the health sector:

‘We have been ignored for a long time, that when opportunities are available, since we work together with you, we desire to have discussions with you, the clinicians. But since I came to this hospital, it’s been three years, and there has never been a meeting between clinicians and lab workers. And when we discuss, we would know the issues, and you would be informed on which machines are faulty to ensure that all the lab investigations you requested are performed.’ (Participant 2, laboratory technician, 20 June 2019)

There is a vibrant private laboratory sector in Freetown, and doctors at MH 34 refer patients to private facilities for diagnostic tests that the MH 34 laboratory cannot perform. Due to the absence of a memorandum of understanding between the MH 34 laboratory and the private laboratories, patients are referred to the private sector, where they usually pay for the services out of pocket and return to the hospital with a printed copy of their results for the attention of the referring clinician. Even though external referrals are viewed by laboratory technicians as justified when the MH 34 laboratory cannot perform such tests, they also perceive clinicians to have poor levels of trust in the quality of their work.

Ethnographic observations and informal interactions outside of the formal interview context suggested a pervasive sense, reported among laboratory workers, that clinicians did not sufficiently value their expertise. It was also difficult to demonstrate the quality of their work when supply chain and infrastructural challenges prevented them from carrying out tests. Regardless of the challenges, laboratory staff reported going the extra mile to process and report urgent results to doctors quickly:

‘Like when we are done in the laboratory, especially if the doctor needs an urgent result like Hb [*haemoglobin*] level of the patient, immediately we’re done we should make sure that the clinician receives the results. If it’s a ward patient, we make sure that we take the results urgently and tell the nurse on duty that, hmm, this patient is alarming; please let the doctor know urgently.’ (Participant 3, laboratory lead, 22 June 2019)

The laboratory staff interviewed, especially the volunteers, also requested improved pay and working conditions:

‘The doctors should be aware that the lab technicians are exposed to much risk in their work setting. We are exposed to several viruses and if we get sick, we expose our family members. We, therefore, need to be given incentives to work well. … I have advocated so they can increase my salary, but that has not come to effect even after repeatedly reminding my superiors. I have worked for over a year, and they have refused to add to my salary. However, other colleagues, such as the cleaners, are receiving more pay than myself, including the electrician. I am distressed to learn that the electrician gets more pay than me, who works as a laboratory technician. I work very hard but get a little incentive in return. Now I am even thinking of getting a second job.’ (Participant 6, laboratory technician, 11 September 2019)

## Discussion

Our study findings show that laboratory staff hold positive valuations of their laboratory work. However, laboratory technicians report feeling undervalued by policymakers and government officials. Despite an increased focus on laboratories during the Ebola crisis, laboratory workers do not view this attention as translating into sustainable progress within the sector, and feel their insights are overlooked when making critical decisions. This perception of neglect is exacerbated by inadequate training opportunities and a lack of investment in laboratory infrastructure, contributing to low morale among laboratory technicians.

Positive perceptions of laboratory work by family members contributed to an understanding of their work as valuable and important, both for the family and for society. This positive sense of the value of their work had, in many cases, been enhanced by their experience of the Ebola outbreak, in which laboratory workers perceived testing had played a crucial role in saving lives and bringing the outbreak to an end. Our finding that laboratory workers had a strong sense of their work’s important contribution to the health sector confirms findings from elsewhere in West Africa that laboratory workers refer to themselves as the ‘brains’ of the healthcare system,^17^ and as the ‘sorcerers’ of the clinicians, who investigate the underlying problem causing their patients’ illness.^19^

However, this sense of pride in their expertise and positive view of their work was contradicted by perceptions of how others viewed it. Our thematic analysis showed that laboratory workers often feel undervalued and under-recognised within the Sierra Leone health system, results that mirror qualitative research on laboratory workers in other institutions in the country^18,19^ and align with research that has shown that laboratories have been historically neglected within health system strengthening efforts in Africa.^26,27^ For the laboratory workers included in this study, the lack of investment in the laboratory infrastructure and equipment, limited career growth opportunities, and poor pay and working conditions evidenced how poorly the government valued their work. The fact that they had little input in the procurement of equipment, were not trained to maintain the equipment they received, and did not receive sufficient supplies to carry out basic tests led to a sense among laboratory staff that their views and experiences were not seen as essential and that little attention was being paid to the logistical and infrastructural systems that enable laboratories to function. The neglect of laboratory infrastructure in the main laboratory was contrasted with how the Chinese government was viewed as valuing and resourcing the work undertaken in the dedicated infectious disease laboratory.

Beyond their relationship with the government, laboratory staff also perceived the laboratory as a neglected space within the hospital. The fact that many clinicians sent patients to private laboratories rather than the hospital’s main laboratory for testing demonstrated the poor regard their clinical colleagues had for them. These tensions echo findings from the laboratory-clinical interface elsewhere in Africa.^28,29^ In Tanzania, for example, a study found that in-person contact between clinicians and laboratory professionals is seldom institutionalised, and collaboration is rare.^30^ As other scholars have shown, relationships between clinicians and laboratory workers must be understood regarding the socio-political, economic, and cultural context in which staff operate.^31^ Our findings suggest that in Sierra Leone, these relationships and a mutual understanding of one another’s professional viewpoints are undermined by the lack of investment in infrastructure, supply chains and human resources.

One key issue raised was the appropriateness of donated laboratory equipment and the establishment of supply systems, training, and maintenance to ensure that those machines continued to be usable in the future. The hierarchical nature of public institutions like the military often leads to decisions being made by top-ranking officials from the Ministry of Defence rather than consulting the laboratory technicians who are familiar with the day-to-day operational needs. Also, it is common for institutions like the Military Hospital to be approached by non-profits, corporate entities, or foreign government agencies with an offer to donate equipment. Structures and systems to vet these donations and determine whether the items given meet the actual needs and conditions of the receiving institution are non-existent. Selection of donated equipment, therefore, does not typically undergo a systematic evaluation to ensure compatibility with existing infrastructure, availability of necessary supplies, and the hospital staff’s ability to operate and maintain the equipment effectively. Another issue was the perceived resource inequality between the ‘main laboratory’ supported by the Sierra Leone Military and the infectious disease laboratory financed by the Chinese military. As others have shown, post-Ebola laboratory-strengthening efforts have often resulted in donations of high-end equipment, including more automated diagnostic devices, and infrastructural investments. But rather than integrating those investments within existing laboratory structures, separate units and parallel vertical systems have often been set up, which fall into disrepair after donors leave.^3^ Our findings show that laboratory staff are acutely aware of these dynamics and that they impact their perception of how their work is valued by others.

Overall, across a wide range of areas, including career and training opportunities, procurement processes, infrastructure investment, clinician-laboratory interactions, laboratory staff described their work as ignored, underpaid, undervalued, and neglected. We suggest that these experiences are best understood as a form of social invisibility in which laboratory workers perceive that the value of their contribution to society is not validated and recognised by others, whether that be fellow hospital staff, managers or government.^32^ We found that social invisibility may be experienced through spatial isolation within the laboratory, lack of consultation on decisions, or physical neglect as when staff work amidst dilapidated infrastructure. This finding that social invisibility is a key aspect of the post-Ebola laboratory worker experience in MH 34 confirms findings from other studies in the region about experiences of hidden labour and undervalued contributions to the response.^14,15,16,17,18,19^ It also mirrors the findings from a qualitative study of healthcare workers in Sierra Leone, which found that a lack of recognition for healthcare providers by superiors and the health system in general led to poor morale and contributed to poor care.^33^

Perhaps the most striking aspect of our findings is that only three years after the end of the Ebola Outbreak, laboratory workers in Sierra Leone see their work as undervalued and unrecognised. Laboratories are increasingly recognised as key to disease control programmes, elimination efforts, and epidemic preparedness and response.^29^ The West Africa Ebola Outbreak was a watershed moment that highlighted the extent to which government and international agencies had overlooked the region’s laboratory systems and ushered in intensive laboratory-strengthening efforts.^1^ Our research shows that the Ebola experience in the career of laboratory workers reinforced their sense of public service and the importance of their work. Nonetheless, laboratory workers expressed disappointment that the highly visible nature of their contribution during Ebola, when laboratory testing was widely recognised as vital to the response, was not reflected in subsequent investments in infrastructure and training. Since these findings were based on a small study with a limited sample size, further research is needed to establish how widely this affected the laboratory sector in the region.

In terms of priorities for policy changes and investments, laboratory workers articulated the need for improvement in training and employment conditions, physical space, infrastructure, restructuring of supply chains and laboratory-driven equipment procurement and maintenance, and the need for improved relationships with clinical staff. The hospital management and the different ministries concerned should consider the alignment and integration of the two laboratories, such as integrating both units as a single ‘main’ laboratory. It is important that these measures are not only viewed as material investments but that they are acknowledged as a key part of the process of social visibility and recognition and as making a potentially key contribution to staff morale.

At MH 34, some of these priorities have already begun to be addressed in response to the findings of this study. In November 2020, the MH 34 initiated a project to enhance laboratory services by identifying and implementing interventions focused on five target areas: staff, supplies, systems, space, and services.^34,35,36^ Most of the interventions directly targeted the concerns raised by laboratory workers as part of this study.

### Staffing

To improve staffing, we recruited additional technicians, data clerks, and laboratory interns, identified gaps in technical competence through regular surveys, and facilitated rotation, in-house training, or collaboration with external partners for specialised training courses.^34,35^

### Supply chains and maintenance

We brokered a public-private partnership with a local laboratory equipment vendor, which allowed us to acquire new equipment, consumables, and reagents with the possibility of deferring payment. We also secured free maintenance for the equipment at the hospital. By shifting from international to local vendors, we achieved a steady and sustainable source of reagents and equipment and broadened our laboratory’s repertoire of services. Our testing capability grew to encompass various fields, such as biochemistry, haematology, hormones, and microscopy, which resulted in a substantial reduction in referrals of inpatients to private laboratories.^35^

### Physical infrastructure

We undertook several renovations to create a more favourable working environment and boost the morale of laboratory technicians. In addition to repainting, we replaced all the air conditioning units, repaired the ceiling to eliminate leaks, increased the water storage capacity to guarantee a reliable water supply, and furnished the on-call room with new furniture, including air conditioning and beds. Other measures we implemented to enhance safety in the workplace included fitting breakers, stabilisers, and inverters (for certain equipment) to safeguard against erratic voltages and ensure optimal performance.^36^

### Workplace relationships

Lastly, we arranged guided tours of the laboratory for hospital clinicians and frequently presented and arranged for laboratory leads to present on aspects of laboratory work at the continuous medical education seminars for hospital staff. Exhibiting the laboratory’s operations and constraints has significantly increased the number of patients that clinicians refer to our laboratory.^35,36^

### Limitations

This research design encompassed several limitations. We acknowledge the limitation imposed by the small sample size. While we sought to mitigate this through a measure of data saturation, the cohort of technician participants represented here may not fully capture the diversity of experiences and perspectives that exist among laboratory workers at large. To safeguard anonymity in a small sample size, we were unable to disclose full demographic and employment details for individual participants. Expanding the sample size in future research could potentially yield more diverse insights and amplify the reliability of the thematic constructs identified.

The study’s setting is limited to a single hospital. While this provides an intimate understanding of the technicians’ experiences within this particular environment, the unique operational and environmental factors specific to this hospital may not be representative of other institutions. Comparison to similar studies, as detailed in the discussion, suggests that the findings have wider applicability. Nonetheless, the findings are best viewed as a detailed case study and further research is needed to establish the extent to which they can be generalised.

While qualitative research inherently involves interpretative elements by the researchers, M.B.J.’s employment at the hospital may introduce additional unconscious biases stemming from their personal experiences and internal knowledge of the hospital environment. To mitigate this, A.S. and E.V. reviewed M.B.J.’s input into the research process and made recommendations for alternative questions, interpretations, and analysis where data collection and data analysis processes were perceived to risk bias. As outlined in the methodology section, steps were also taken to limit participant bias stemming from M.B.J.’s position as a hospital employee. While some bias may nonetheless have persisted, the results indicate that participants felt safe to mention several concerns related to clinicians, the infrastructure, hospital administration and even the way the Ministry of Defence undertakes procurement of supplies.

The highlighted limitations underscore the need for cautious interpretation of the study’s outcomes. To enhance the validity and extend the applicability of the findings from this research, future studies with larger sample sizes, involving multiple hospitals with varying characteristics, and including research multiple team members, including researchers who are not affiliated with the study settings, are recommended.

### Conclusion

The neglected state of laboratories in African healthcare systems has been improving since the Ebola outbreak in West Africa, but maintaining investments after the end of an emergency and the exit of many donors has been a challenge. Laboratories are essential in outbreak response, but this study shows that laboratory workers feel undervalued due to a lack of investment, limited career opportunities, poor pay, pressured working conditions, and clinicians sending patients to private labs.

It is important that laboratory workers’ perceptions and experiences are understood, listened to and acted upon when setting priorities for national and international health system strengthening efforts. Laboratory staff at MH 34 identified investment in training, improved employment conditions, physical space, integration of donor and local infrastructures, equipment maintenance, supply chain restructuring, and clinical staff relationships as key priorities that should be addressed if they are to feel valued and contribute effectively to routine patient care and emergency response. Integrating research findings from this study into laboratory restructuring and investment at MH 34 provides an example of how qualitative research, which focuses on people’s experiences, relationships, and practices, can contribute to strengthening laboratory systems in efficient ways.
